# Multi-view graph-regularized deep metric subspace clustering network

**DOI:** 10.1371/journal.pone.0354307

**Published:** 2026-07-24

**Authors:** Pengpeng Luo, Ming Yang, Chong Peng, Qianqian Wang

**Affiliations:** 1 College of Mathematical Sciences, Harbin Engineering University, Harbin, Heilongjiang, PR China; 2 College of Computer Science and Technology, Ocean University of China, Qingdao, Shandong, PR China; 3 School of Telecommunications Engineering, Xidian University, Xi’an, PR China; Shiv Nadar University, INDIA

## Abstract

Multi-view subspace clustering has progressed significantly by using deep neural networks to handle nonlinear data representations. A recent advancement, the Multi-view Self-Expressive Subspace Clustering (MSESC) network, achieves markedly higher computational efficiency by substituting the traditional self-expression layer with a deep metric learning approach. Nevertheless, MSESC still suffers from two notable limitations: it fails to adequately capture the high-order geometric structures inherent in multi-view data, and it lacks effective guidance from the underlying clustering distribution. To overcome these shortcomings, we propose a novel framework termed Multi-View Graph Regularized Deep Metric Subspace Clustering (MVGR-DMSC). The proposed method introduces two key components into MSESC to enhance the discriminability of representations. First, a dual-order graph regularization module is devised to maintain both first-order and second-order manifold structures, thereby allowing the model to capture more complex local geometric relationships. Second, an adaptive view-weighted deep clustering module is incorporated, which employs the Kullback–Leibler divergence to guide representation learning while dynamically adjusting the contributions of different views. Through evaluations on five benchmark datasets, we show that MVGR-DMSC consistently yields better results than several state-of-the-art approaches, including the direct baseline MSESC, in both accuracy and robustness.

## Introduction

As data collection technology advances, it has become common to gather large-scale information from multiple sensors, modalities, or perspectives. These datasets, known as *multi-view data*, offer a mix of unique view-specific features and shared information that is consistent across different views. Benefiting from this property, multi-view clustering (MVC) has garnered sustained interest within the machine learning and data mining research communities [1,2]. The objective of MVC is to divide unlabeled samples into distinct clusters by jointly leveraging the inherent correlations that exist across different views. Over the last ten years, numerous MVC approaches have been proposed, which are generally classified into four main categories: methods based on nonnegative matrix factorization (NMF) [3], graph-based techniques [4], approaches based on kernel learning, as well as those founded on subspace learning [5,6]. More recently, tensor-based methods have emerged as a powerful paradigm for exploiting high-order correlations across views. For example, ARIA [7] introduces alternative rank-minimizing regularization to achieve a tighter approximation of the tensor rank function, which effectively captures the consistent and complementary information embedded in the multi-view representations.

Within this landscape, Multi-View Subspace Clustering (MVSC) has distinguished itself as a prominent framework due to its strong theoretical foundation in the self-expressiveness property and its superior ability to handle high-dimensional data by identifying the underlying low-dimensional subspaces. The foundation of MVSC is the classical self-expressiveness property, which posits that any given data point can be expressed as a combination, using linear weights, of other points originating from its specific subspace. Guided by this principle, the majority of MVSC techniques are designed to acquire either view-specific or shared coefficients for self-representation. Subsequently, they enforce a form of consensus regularization across the different views, thereby constructing a unified affinity matrix suitable for the final spectral clustering [5,6,8]. Owing to their solid geometric interpretation and strong empirical performance, MVSC methods have been successfully applied in many scenarios. Nevertheless, conventional MVSC models are still limited in two important aspects. First, they usually rely on shallow linear representations and therefore lack sufficient capacity to capture complex nonlinear structures embedded in real-world data. Second, building and optimizing the self-expression layer frequently leads to substantial computational and memory overhead, particularly when dealing with large-scale datasets.

To overcome these limitations of shallow models, some works introduces deep neural networks into multi-view subspace clustering and developed deep multi-view subspace clustering(DMVSC). Existing DMVSC methods typically employ deep autoencoders or convolutional encoders to learn nonlinear transformations for different views and then integrate the learned latent features into a self-expression framework [9–14]. These methods significantly enhance the expressive power of subspace clustering and improve performance on complex data. However, numerous deep subspace clustering methods continue to rely on the costly self-expression layer, the memory complexity of which grows quadratically as the number of samples increases. To further enhance the discriminative power of latent features, some researchers have explored the use of generative adversarial networks. Deep Adversarial Multi-view Clustering (DAMC) [15] leverages adversarial training to capture the complex data distribution of each view, thereby learning a more disentangled and cluster-friendly latent space across multiple perspectives.

To address this issue, Cui *et al.* [16] introduced the Multi-view Self-Expressive Subspace Clustering (MSESC) network, which substitutes a deep metric learning mechanism for the traditional self-expression layer, thereby lowering the memory complexity from $𝒪(N^{2})$ to 𝒪(N). MSESC markedly enhances the scalability of deep multi-view subspace clustering. However, MSESC still exhibits two important limitations that restrict its full potential as follows.

1. **Insufficient exploitation of complex manifold structures.** MSESC mainly emphasizes global metric-based similarity learning and only employs a relatively simple first-order graph constraint to preserve local structure. As a result, high-order geometric relationships are not adequately explored. Nevertheless, these high-order manifold structures are frequently essential for depicting the intrinsic geometry of complex multi-view data and for enhancing clustering discriminability [17,18]. The potential of high-order relationships has also been demonstrated in other domains; for instance, DIVIDE [19] utilizes high-order random walks within a contrastive learning framework to progressively identify potential positive samples from high-order neighbors. This underscores the necessity of moving beyond simple first-order graph constraints to capture more intricate local geometric structures.
2. **Lack of adaptive clustering-oriented guidance.** MSESC largely treats representation learning and cluster structure refinement as loosely coupled processes, and thus fails to fully utilize the intrinsic clustering distribution to guide feature learning. In addition, it implicitly assumes that all views contribute equally to the final clustering result, which may be suboptimal in practice because different views often have unequal discriminative abilities and noise levels.

**Fig 1:**
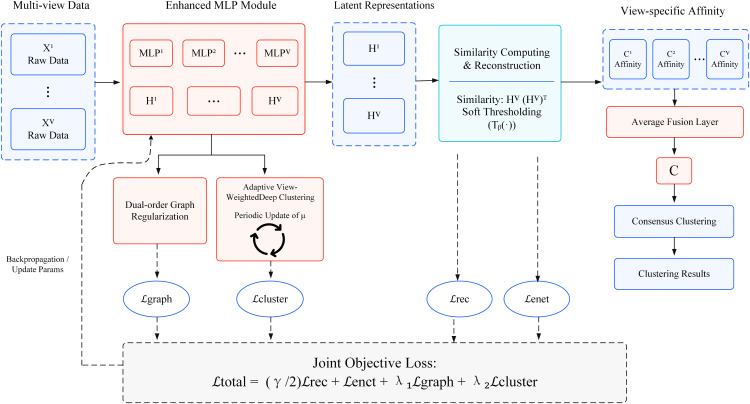
An overview of the proposed MVGR-DMSC architecture. The model is built upon four key components: (1) an **Enhanced MLP Module** that extracts latent representations from raw multi-view inputs; (2) a **Similarity Computing & Reconstruction** module which constructs the affinity matrix via inner-product similarity and adaptive soft-thresholding; (3) a **Dual-order Graph Regularization** module designed to maintain high-order local manifold structures; and (4) an **Adaptive View-weighted Deep Clustering** module that leverages KL divergence to steer the feature learning process adaptively. Ultimately, the view-specific affinity matrices are integrated through an average fusion layer, yielding a consensus affinity matrix **C** that serves as the basis for the final clustering.

To tackle the aforementioned challenges, we present a straightforward yet comprehensive and potent framework, designated as *Multi-View Graph Regularized Deep Metric Subspace Clustering* (MVGR-DMSC). The complete framework of our presented approach is depicted in Fig 1. Built upon the efficient deep metric learning architecture of MSESC, MVGR-DMSC further strengthens representation learning from two complementary perspectives.

The proposed framework incorporates two complementary strategies to enhance representation learning. First, we employ a dual-order graph regularization approach that simultaneously preserves both first-order and second-order neighborhood structures within the latent space. This design enables the model to capture more intricate local geometric relationships and more accurately reflect the underlying manifold of multi-view data. Second, drawing inspiration from deep clustering techniques such as DEC [20], we develop an adaptive view-weighted clustering module. This module aligns the soft assignment distribution with a sharpened target distribution through Kullback–Leibler (KL) divergence, thereby introducing clustering-oriented guidance into the feature learning process. Simultaneously, it adaptively weights the contributions from different views, mitigating the impact of low-quality or less informative views. By unifying deep metric similarity learning, dual-order graph structure preservation, and clustering-distribution guidance within a single optimization framework, MVGR-DMSC produces a more discriminative and robust consensus affinity matrix, leading to improved clustering outcomes.

The main contributions of this paper are as follows:

- We propose a novel deep multi-view subspace clustering framework termed MVGR-DMSC. Building on the efficient metric learning architecture of MSESC, our approach enhances clustering performance through improved geometric structure preservation and clustering-aware representation learning.
- We integrate a dual-order graph regularization module into the deep multi-view subspace clustering framework. By leveraging both first-order and second-order graph Laplacian regularizations, our method effectively preserves the complex local manifold structures characteristic of multi-view data.
- We design an adaptive view-weighted deep clustering module that leverages clustering distributions to steer feature learning while adaptively assessing the relative importance of each view. This strategy results in a more discriminative and robust consensus affinity matrix.
- Extensive experiments on five benchmark multi-view datasets demonstrate that MVGR-DMSC consistently outperforms various state-of-the-art methods, including its direct predecessor MSESC, in terms of both clustering accuracy and robustness.

## Related work

This section briefly surveys the literature most relevant to our approach, with a particular focus on three key areas: multi-view subspace clustering, its deep extension, and graph-regularized deep clustering techniques.

### Multi-view subspace clustering

The self-expressiveness model serves as the theoretical foundation for subspace clustering. It posits that any data point lying in a union of subspaces can be expressed as a linear combination of other points originating from the same subspace [21–25]. Given a data matrix $𝐗\in {\mathbb{R}}^{D\times N}$, this principle leads to the following optimization problem for learning a coefficient matrix $𝐂\in {\mathbb{R}}^{N\times N}$:


$$\begin{array}{c} \min_{𝐂}\|𝐂{\|}_{p}+\lambda \|𝐗-𝐗𝐂{\|}_{F}^{2},\;\text{s.t. }diag(𝐂)=0, \end{array}$$ (1)

where $\|\cdot {\|}_{p}$ typically denotes a norm that encourages sparsity (e.g., $\ell_{1}$) or low-rank structure (e.g., nuclear norm). Two representative approaches following this paradigm are sparse subspace clustering (SSC) [21] and low-rank representation (LRR) [22], both of which have proven effective at recovering latent subspace structures.

When multiple feature representations are available, multi-view subspace clustering seeks to leverage both the shared information and view-specific characteristics. Specifically, given a multi-view dataset $\{{𝐗}^{\left(v\right)}{\}}_{v=1}^{V}$, A widely adopted strategy first constructs view-specific self-representation matrices $\{{𝐂}^{\left(v\right)}{\}}_{v=1}^{V}$, and then integrates them into a unified affinity graph for subsequent spectral clustering [6,26,27]. We can formalize this procedure as:


$$\begin{array}{c} \min_{\{{𝐂}^{\left(v\right)}\},{𝐂}^{*}}\sum_{v=1}^{V}\left(\|{𝐗}^{\left(v\right)}-{𝐗}^{\left(v\right)}{𝐂}^{\left(v\right)}{\|}_{F}^{2}+\alpha \|{𝐂}^{\left(v\right)}-{𝐂}^{*}{\|}_{F}^{2}\right)+\beta \|{𝐂}^{*}{\|}_{p}. \end{array}$$ (2)

To capture higher-order relationships across views, recent research has explored tensor-based formulations. These methods arrange the view-specific representation matrices as a third-order tensor and enforce low-rank constraints in the tensor domain, thereby aiming to model complex inter-view correlations more explicitly [26,28–33]. However, despite their ability to handle linear dependencies, such shallow models often fall short when confronted with the nonlinear structures prevalent in real-world multi-view data.

### Deep multi-view subspace clustering

Deep subspace clustering methods tackle the limitations inherent in shallow linear models by employing neural networks to learn nonlinear latent features that embody the self-expressiveness property [34–38]. A typical design employs an encoder to map input data into latent representations ${𝐇}^{\left(v\right)}$; afterwards, a self-expression layer takes these features and reconstructs each latent code using other codes from the same view. The training objective typically comprises both a reconstruction term and a self-expression regularization component:


$$\begin{array}{c} ℒ=\sum_{v=1}^{V}\left(\|{𝐗}^{\left(v\right)}-{\hat{𝐗}}^{\left(v\right)}{\|}_{F}^{2}+\gamma \|{𝐇}^{\left(v\right)}-{𝐇}^{\left(v\right)}{𝐂}^{\left(v\right)}{\|}_{F}^{2}\right)+\Omega ({𝐂}^{\left(v\right)}). \end{array}$$ (3)

By jointly optimizing feature learning and affinity construction in an end-to-end manner, this approach substantially boosts the representational quality and clustering accuracy.

More recently, this concept has been expanded to the multi-view setting, wherein multiple encoders are employed to extract view-specific features, and a shared or coordinated self-representation mechanism is utilized to capture inter-view consistency [10]. Although these methods achieve promising results, the majority of current deep multi-view subspace clustering approaches continue to depend on an explicit self-expression layer, the parameter size of which grows as $𝒪(N^{2})$ relative to the sample count. This leads to considerable memory and computational overhead, especially for large-scale datasets.

To alleviate this issue, Cui *et al.* proposed the Multi-view Self-Expressive Subspace Clustering (MSESC) network [16], which replaces the explicit self-expression layer with a deep metric learning mechanism and constructs affinities through feature similarities. This design significantly improves scalability and efficiency. Nevertheless, MSESC mainly emphasizes pairwise metric relations and simple neighborhood consistency, while paying insufficient attention to high-order geometric dependencies and clustering-oriented distribution refinement. In contrast, our method augments the MSESC architecture with dual-order graph regularization and adaptive deep clustering, thereby enhancing both geometric structure preservation and discriminative representation learning.

### Graph regularization and deep clustering

Graph regularization has found extensive application in representation learning and clustering for maintaining the local manifold structure of data [39,40]. The underlying principle holds that samples which are adjacent in the original space ought to stay proximate within the learned latent space. A commonly used regularizer is the Laplacian term


$$\begin{array}{c} Tr(𝐇𝐋{𝐇}^{⊤}), \end{array}$$ (4)

where **L** denotes the graph Laplacian constructed from an affinity graph. This regularization has been successfully incorporated into many shallow and deep clustering methods to improve local structure preservation [41–43]. Nevertheless, the majority of current approaches concentrate mainly on first-order neighborhood information and often struggle to capture more intricate high-order geometric relationships.

Another important line of research is deep clustering with distribution refinement. Representative methods, such as DEC [44] and IDEC [41], iteratively refine latent representations by matching soft cluster assignments to an auxiliary target distribution through the Kullback–Leibler (KL) divergence. This strategy promotes tighter intra-cluster formations and more distinct separations between cluster boundaries. Subsequent studies have shown that distribution-guided clustering objectives can substantially enhance the discriminability and robustness of learned features [41,44].

Although graph regularization and distribution-based deep clustering have both achieved notable success, their effective integration into efficient deep multi-view subspace clustering remains insufficiently explored. In particular, there is still a lack of a unified framework that can simultaneously preserve first-order and second-order manifold structures while adaptively weighting the contributions of different views during clustering. This gap motivates the proposed Multi-View Graph Regularized Deep Metric Subspace Clustering (MVGR-DMSC).

## Proposed method

### Problem formulation

As introduced previously, Let $\{{𝐗}^{\left(v\right)}\in {\mathbb{R}}^{N\times d_{v}}{\}}_{v=1}^{V}$ denote a multi-view dataset, where *V* is the number of views, *N* is the number of samples, and $d_{v}$ is the dimensionality of the *v*-th view. The goal is to partition the samples into *K* clusters by learning discriminative latent representations together with a consistent multi-view clustering structure.

Current deep subspace clustering approaches commonly introduce a self-expression layer to learn an explicit N×N coefficient matrix, which incurs high memory and computational costs. Moreover, they often ignore higher-order geometric structures and do not explicitly exploit the intrinsic clustering distribution during representation learning. To tackle these problems, we introduce *Multi-View Graph Regularized Deep Metric Subspace Clustering* (MVGR-DMSC) framework, which integrates metric-based self-representation, dual-order graph regularization, and adaptive view-weighted deep clustering into a unified model.

### View-specific nonlinear embedding

For each view, we learn a nonlinear mapping


$$\begin{array}{c} {𝐇}^{\left(v\right)}=f_{\theta^{\left(v\right)}}({𝐗}^{\left(v\right)})\in {\mathbb{R}}^{N\times d}, \end{array}$$ (5)

where $f_{\theta^{\left(v\right)}}(\cdot )$ is a view-specific MLP consisting of *l* fully connected layers with ReLU activations, and *d* is the common latent dimension. A tanh activation is imposed at the output layer to bound the latent codes, which stabilizes the subsequent similarity computation and suppresses scale variation across views.

### Metric-based self-representation

Rather than learning an explicit self-expression matrix, we directly obtain the affinity coefficients from the latent representations. For the *v*-th view, the coefficient matrix takes the form


$$\begin{array}{c} {𝐂}^{\left(v\right)}={𝒯}_{\beta^{\left(v\right)}}\;\left({𝐇}^{\left(v\right)}({𝐇}^{\left(v\right)}{)}^{⊤}\right), \end{array}$$ (6)

where ${𝒯}_{\beta }(\cdot )$ denotes the elementwise soft-thresholding operator


$$\begin{array}{c} {𝒯}_{\beta }(z)=sgn(z)\max(0,|z|-\beta ), \end{array}$$ (7)

and $\beta^{\left(v\right)}\geq 0$ is a learnable threshold.

This approach eliminates the need for an extra trainable N×N layer and naturally yields sparse affinities. The reconstructed data matrix is computed as


$$\begin{array}{c} {\hat{𝐗}}^{\left(v\right)}=({𝐂}^{\left(v\right)}-Diag({𝐂}^{\left(v\right)})){𝐗}^{\left(v\right)}, \end{array}$$ (8)

which imposes the conventional zero-diagonal constraint and avoids trivial self-reconstruction. The reconstruction loss is given by


$$\begin{array}{c} {ℒ}_{rec}=\sum_{v=1}^{V}{\left\|{𝐗}^{\left(v\right)}-{\hat{𝐗}}^{\left(v\right)}\right\|}_{F}^{2}. \end{array}$$ (9)

To regularize the learned affinities, we incorporate an Elastic Net penalty:


$$\begin{array}{c} {ℒ}_{enet}=\sum_{v=1}^{V}\left(\lambda \|{𝐂}^{\left(v\right)}{\|}_{1}+\frac{1-\lambda }{2}\|{𝐂}^{\left(v\right)}{\|}_{F}^{2}\right), \end{array}$$ (10)

where the $\ell_{1}$ term encourages sparsity, while the Frobenius term promotes the grouping effect. After obtaining the view-specific affinity matrices $\{{𝐂}^{\left(v\right)}{\}}_{v=1}^{V}$, the consensus affinity matrix **C** is computed by averaging them across all views, i.e., $𝐂=\frac{1}{V}\sum_{v=1}^{V}{𝐂}^{\left(v\right)}$. Finally, spectral clustering is performed on **C** to generate the final clustering results.

### Dual-order graph regularization

To maintain both local adjacency and higher-order neighborhood relationships, we build first-order and second-order graphs in the latent space. Let ${𝐀}^{\left(v\right)}$ be the *k*-nearest-neighbor graph derived from ${𝐇}^{\left(v\right)}$. We then define


$$\begin{array}{c} {𝐖}_{1}^{\left(v\right)}={𝐀}^{\left(v\right)},\;{𝐖}_{2}^{\left(v\right)}={𝐀}^{\left(v\right)}({𝐀}^{\left(v\right)}{)}^{⊤}. \end{array}$$ (11)

The corresponding normalized Laplacian matrices are computed as


$$\begin{array}{c} {𝐋}_{k}^{\left(v\right)}=𝐈-({𝐃}_{k}^{\left(v\right)}{)}^{-1/2}{𝐖}_{k}^{\left(v\right)}({𝐃}_{k}^{\left(v\right)}{)}^{-1/2},\;k\in \{1,2\}, \end{array}$$ (12)

where ${𝐃}_{k}^{\left(v\right)}$ denotes the degree matrix of ${𝐖}_{k}^{\left(v\right)}$.

Based on the Laplacian matrices, we further construct the graph regularization term as follows:


$$\begin{array}{c} {ℒ}_{graph}=\sum_{v=1}^{V}\sum_{k=1}^{2}Tr(({𝐇}^{\left(v\right)}{)}^{⊤}{𝐋}_{k}^{\left(v\right)}{𝐇}^{\left(v\right)}). \end{array}$$ (13)

Since each ${𝐋}_{k}^{\left(v\right)}$ is positive semidefinite, ${ℒ}_{graph}$ is always nonnegative. Furthermore, this quadratic form penalizes significant variations in representations along graph edges, thereby encouraging manifold smoothness across both direct and indirect sample connections.

Specifically, the first-order graph ${𝐖}_{1}^{\left(v\right)}$ focuses on direct adjacency, ensuring that immediate neighbors in the original space remain proximate in the latent space to maintain local smoothness. Conversely, the second-order graph ${𝐖}_{2}^{\left(v\right)}$ captures contextual similarity by considering shared neighborhood structures—essentially identifying whether two points share the same neighbors. This allows the model to maintain manifold consistency even when direct connections are weak or corrupted by noise. By integrating both orders, MVGR-DMSC can simultaneously preserve fine-grained local details and the broader geometric topology, which significantly enhances the discriminability of the learned representations compared to models using only simple first-order constraints.

### Adaptive view-weighted deep clustering

To enhance cluster separability, we introduce a KL-based deep clustering module. Let ${𝐡}_{i}^{\left(v\right)}$ denote the *i*-th row of the latent representation matrix ${𝐇}^{\left(v\right)}$. For the *v*-th view, the soft assignment is defined by


$$\begin{array}{c} q_{ij}^{\left(v\right)}=\frac{{\left(1+\|{𝐡}_{i}^{\left(v\right)}-\mu_{j}^{\left(v\right)}{\|}_{2}^{2}/\alpha \right)}^{-\frac{\alpha +1}{2}}}{\sum_{k=1}^{K}{\left(1+\|{𝐡}_{i}^{\left(v\right)}-\mu_{k}^{\left(v\right)}{\|}_{2}^{2}/\alpha \right)}^{-\frac{\alpha +1}{2}}}, \end{array}$$ (14)

where $\{\mu_{j}^{\left(v\right)}{\}}_{j=1}^{K}$ are the cluster centers in the latent space and α is a fixed parameter representing the degrees of freedom. The sharpened target distribution is


$$\begin{array}{c} p_{ij}^{\left(v\right)}=\frac{q_{ij}^{(v)2}/f_{j}^{\left(v\right)}}{\sum_{k=1}^{K}q_{ik}^{(v)2}/f_{k}^{\left(v\right)}},\;f_{j}^{\left(v\right)}=\sum_{i=1}^{N}q_{ij}^{\left(v\right)}. \end{array}$$ (15)

Let ${𝐪}_{i}^{\left(v\right)}=[q_{i1}^{\left(v\right)},\ldots ,q_{iK}^{\left(v\right)}{]}^{⊤}$ denote the soft assignment vector for the *i*-th sample in the *v*-th view. To adaptively exploit view complementarity, we learn sample-wise view weights through


$$\begin{array}{c} {𝐰}_{i}=Softmax(g_{\phi }([{𝐪}_{i}^{\left(1\right)},\ldots ,{𝐪}_{i}^{\left(V\right)}])), \end{array}$$ (16)

where ${𝐰}_{i}=[w_{i}^{\left(1\right)},\ldots ,w_{i}^{\left(V\right)}{]}^{⊤}$ satisfies $w_{i}^{\left(v\right)}\geq 0$ and $\sum_{v=1}^{V}w_{i}^{\left(v\right)}=1$. The clustering loss is then


$$\begin{array}{c} {ℒ}_{cluster}=\sum_{v=1}^{V}\sum_{i=1}^{N}w_{i}^{\left(v\right)}\sum_{j=1}^{K}p_{ij}^{\left(v\right)}\log\frac{p_{ij}^{\left(v\right)}}{q_{ij}^{\left(v\right)}}. \end{array}$$ (17)

This term explicitly drives the learned representations toward compact and confident cluster assignments.

### Overall objective

The final objective function is


$$\begin{array}{c} ℒ=\frac{\gamma }{2}{ℒ}_{rec}+{ℒ}_{enet}+\lambda_{1}{ℒ}_{graph}+\lambda_{2}{ℒ}_{cluster}, \end{array}$$ (18)

where γ>0, $\lambda_{1}>0$, and $\lambda_{2}>0$ are trade-off parameters.

The four terms in (18) play complementary roles: ${ℒ}_{rec}$ preserves self-expressiveness, ${ℒ}_{enet}$ regularizes the affinity structure, ${ℒ}_{graph}$ preserves geometric smoothness, and ${ℒ}_{cluster}$ improves clustering discriminability. Since all terms are nonnegative, ℒ is lower bounded by zero, which provides a basic guarantee for stable optimization.

### Optimization

The entire network is trained in an end-to-end manner using the Adam. The learnable parameters include the encoder parameters $\{\theta^{\left(v\right)}{\}}_{v=1}^{V}$, the threshold parameters $\{\beta^{\left(v\right)}{\}}_{v=1}^{V}$, and the adaptive weighting parameters ϕ. A cosine annealing schedule is adopted for learning rate decay.

We initialize the cluster centers by applying K-means to the initial latent features and periodically update them throughout the training process. Unlike ADMM-based self-expression models, our method computes the coefficient matrices directly through forward propagation, converting the large-scale matrix optimization problem into a standard mini-batch training procedure. Traditional self-expression based methods generally require solving optimization problems with a complexity of $𝒪(N^{2})$ or $𝒪(N^{3})$ per iteration, where *N* is the number of samples. In contrast, for a mini-batch of size *m* and latent dimension *d*, the dominant cost of our metric-based similarity computation is $𝒪(m^{2}d)$ per view. Since m≪N, the proposed framework has significantly lower memory overhead and is substantially more scalable than conventional deep self-expressive clustering methods on large datasets. In our experiments, the model typically converges within a few minutes on a single GPU, which is much faster than the time required by traditional subspace clustering algorithms.

**Algorithm 1 The optimization of MVGR-DMSC**.

**Input:** Multi-view dataset $\{{𝐗}^{\left(v\right)}{\}}_{v=1}^{V}$, number of clusters *K*, trade-off parameters γ (reconstruction weight), $\lambda_{\text{enet}}$ (elastic net balance), $\lambda_{\text{graph}}$ (graph regularization weight), $\lambda_{\text{cluster}}$ (clustering loss weight), number of epochs *T*.

**Output:** Trained network parameters Θ and the consensus affinity matrix **C**.

1:  **Initialization:** Learning rate *lr* = 10^−4^, randomly initialize network parameters Θ. Initialize cluster centers $\{\mu_{k}^{\left(v\right)}{\}}_{k=1}^{K}$ via K-means.

2:  **for** t=1,…,T **do**

3:   Shuffle the dataset and divide it into mini-batches.

4:   **for** each mini-batch **do**

5:    **for** each v∈{1,…,V} **do**

6:     Compute latent representation ${𝐇}^{\left(v\right)}$ via MLP.

7:     Compute soft assignment ${𝐪}^{\left(v\right)}$ and target distribution ${𝐩}^{\left(v\right)}$.

8:     Construct Laplacian matrices ${𝐋}_{1}^{\left(v\right)}$ and ${𝐋}_{2}^{\left(v\right)}$.

9:     Compute ${𝐂}^{\left(v\right)}=\text{SoftThreshold}\left({𝐇}^{\left(v\right)}({𝐇}^{\left(v\right)}{)}^{⊤},\beta \right)$.

10:    Compute reconstructed data ${\hat{𝐗}}^{\left(v\right)}$.

11:   **end for**

12:   Compute adaptive view weights *w*^(*v*)^ via Adaptive Weight Module.

13:   Calculate the overall objective loss ℒ according to Eq. (18).

14:   Update network parameters Θ by gradient descent (Adam).

15:  **end for**

16:  **if** t(mod10)==0 **then**

17:   Update cluster centers $\{\mu_{k}^{\left(v\right)}{\}}_{k=1}^{K}$ via K-means on the whole dataset.

18:  **end if**

19: **end for**

20: **Output:** Perform spectral clustering on the consensus affinity matrix **C** to obtain the final clustering results.

## Experiments

### Datasets

To comprehensively assess the efficacy of the proposed MVGR-DMSC method, we carry out experiments on five commonly used multi-view benchmark datasets. These include three text collections (ACM, Cora, Wikipedia) and two image datasets (Caltech101_20, Handwritten). Detailed statistics for these datasets are provided in Table 1.

**Table 1:** Summary statistics of the multi-view datasets used in our experiments.

Datasets	Type	Samples	Views	Clusters
ACM	Text	3025	5	3
Cora	Text	2708	4	7
Wikipedia	Text	2866	2	10
Caltech101_20	Image	2386	6	20
Handwritten	Image	2000	6	10

### Compared clustering algorithms

To rigorously evaluate the performance of our proposed MVGR-DMSC, we adopt several representative clustering algorithms as baselines, encompassing both traditional approaches and recent deep multi-view clustering techniques. In addition to the methods considered in the original MSESC work, we further incorporate a number of contemporary deep multi-view clustering models to ensure a comprehensive comparison. The complete set of compared methods is listed below:

- **Best K-means** and **Best SC**: Classic single-view clustering methods performed on the best-performing single view.
- **BMVC** [45]: A binary multi-view clustering approach that learns per-view hash codes.
- **LMVSC** [46]: A method for large-scale multi-view subspace clustering that fuses view-specific graphs.
- **MSCNGL** [47]: A multi-view subspace clustering network leveraging both local and global graph information.
- **SDSNE** [48]: A co-supervised strategy that incorporates structural information.
- **MFLVC** [49]: Multi-level feature learning for contrastive multi-view clustering.
- **DSMVC** [50]: A deep safe multi-view clustering framework designed to reduce performance degradation caused by increasing views.
- **MSESC** [16]: A multi-view self-expressive subspace clustering network that serves as the direct baseline for our work.
- **CVCL** [51]: Deep multiview clustering by contrasting cluster assignments.
- **DDMVC** [52]: Deep multi-view clustering with diverse and discriminative feature learning.
- **GCFAggMVC** [53]: Global and cross-view feature aggregation for multi-view clustering.
- **MSCIB** [54]: Multi-view semantic consistency based information bottleneck for clustering.
- **PNCL-FDMC** [55]: Progressive neighbor-masked contrastive learning for fusion-style deep multi-view clustering.

### Experimental setup

We conduct all experiments on a Windows 11 platform equipped with an NVIDIA RTX 5060Ti GPU (16GB memory).

For the network architecture, each view-specific MLP $f_{\theta^{\left(v\right)}}$ consists of four fully connected layers with hidden dimensions of 1024, and the final latent dimension *d* is also set to 1024. We use a batch size of 2386 for mini-batch training. Regarding the deep clustering module, the parameter α in Eq. (14) is set to 1.0, following the standard setting in t-distribution based clustering. All trade-off parameters in the objective function are determined via the grid search strategy described in the Parameter Analysis section.

For clustering performance evaluation, we adopt three commonly used metrics: unsupervised clustering Accuracy (ACC), Normalized Mutual Information (NMI), and Adjusted Rand Index (ARI). For each metric, higher values reflect better clustering quality. **We note that our focus is on comparing the effectiveness of the regularizers, rather than on achieving state-of-the-art results on the benchmark datasets. To this end, we perform thorough ablation studies to isolate and measure the contribution of each regularization component.**

### Experimental results and analysis

To verify the effectiveness of our presented method, we perform two sets of experiments. First, we compare MVGR-DMSC against a variety of state-of-the-art methods on the Caltech101−20 dataset, where the results of the baseline methods are directly taken from the original MSESC paper [16]. We attempted to reproduce these results but found that the reported metrics could not be matched exactly; therefore, we rely on the published values to ensure a fair comparison. Second, we provide comprehensive comparisons on four additional multi-view datasets (ACM, Cora, Wikipedia, Handwritten) against multiple recent deep clustering approaches. For these datasets, all comparative results are obtained by executing the publicly released code provided by the authors, with identical experimental configurations to ensure fairness.

As presented in Table 2, MVGR-DMSC attains the highest performance across all three metrics on Caltech101−20, surpassing both conventional methods and recent deep clustering approaches. This demonstrates the effectiveness of our proposed architecture.

**Table 2:** Clustering results on Caltech101−20 dataset.

Datasets	Caltech101−20		
Evaluation	ACC	NMI	ARI
Best K-means	0.4614	0.6446	0.3363
Best SC	0.5704	0.5769	0.4344
BMVC	0.3923	0.3975	0.2190
LMVSC	0.3508	0.2990	0.1577
MSCNGL	0.4585	0.5526	0.3446
SDSNE	0.5419	0.6302	0.1577
DSMVC	0.3407	0.4574	0.2334
MSESC	0.5277	0.6620	0.4454
**MVGR-DMSC(Our)**	**0.5855**	**0.6841**	**0.4906**

To further evaluate the generalization ability of MVGR-DMSC, we compare it with a broader set of recent methods on three text datasets (ACM, Cora, Wikipedia) and one image dataset (Handwritten). The experimental outcomes are summarized in Tables 3 and 4. For each metric, the optimal result is indicated in boldface.

**Table 3:** Clustering results on text datasets (ACM, Cora, Wikipedia).

Datasets	ACM			Cora			Wikipedia		
Evaluation	ACC	NMI	ARI	ACC	NMI	ARI	ACC	NMI	ARI
CVCL	0.3493	0.0000	0.0000	0.3256	0.1169	0.0796	0.5039	0.4069	0.3122
DDMVC	0.6063	0.2909	0.3037	0.3534	0.0588	0.0529	**0.5454**	0.4371	0.3597
GCFAggMVC	0.5048	0.0838	0.0924	0.2275	0.0249	0.0180	0.5056	0.4636	0.3252
MSCIB	**0.7312**	**0.4606**	**0.4211**	0.3061	0.0564	0.0403	0.5122	0.4029	0.3110
MFLVC	0.6559	0.4255	0.3605	0.2718	0.0567	0.0207	0.4421	0.3825	0.2796
PNCL-FDMC	0.6476	0.4073	0.3409	0.3139	0.1394	0.0622	0.5234	0.4908	0.3226
MSESC	0.3537	0.2779	0.1936	0.3028	0.2245	0.0732	0.3562	0.2966	0.2117
**MVGR-DMSC(Our)**	0.4952	0.3772	0.3244	**0.3634**	**0.2916**	**0.1567**	0.4627	**0.5175**	**0.3622**

**Table 4:** Clustering results on image datasets (Caltech101_20, Handwritten).

Datasets	Caltech101_20			Handwritten		
Evaluation	ACC	NMI	ARI	ACC	NMI	ARI
CVCL	0.3404	0.5494	0.2376	0.8020	0.7808	0.7041
DDMVC	0.5457	0.4348	**0.5256**	**0.9605**	**0.9170**	**0.9151**
GCFAggMVC	0.2175	0.2228	0.0799	0.5715	0.5227	0.3999
MSCIB	0.4061	0.4545	0.2811	0.7760	0.7365	0.6399
MFLVC	0.4593	0.5904	0.4209	0.8700	0.8810	0.8277
PNCL-FDMC	0.3734	0.5798	0.2900	0.7550	0.7306	0.6155
MSESC	0.5277	0.6620	0.4454	0.7580	0.8521	0.7642
**MVGR-DMSC(Our)**	**0.5855**	**0.6841**	**0.4906**	0.7840	0.8758	0.8112

Looking at the results in Tables 3 and 4, we can make several observations. First, compared to its direct baseline MSESC, our MVGR-DMSC achieves consistent and substantial improvements across all four datasets. The most notable gains are observed on the Wikipedia dataset, where our method improves ACC from 0.356 to 0.463 (over 11% increase), NMI from 0.297 to 0.517 (a remarkable 22% improvement), and ARI from 0.212 to 0.362 (a 15% gain). These significant leaps strongly validate that the dual-order graph regularization and adaptive view-weighted clustering effectively capture more complex data structures and learn more discriminative representations.

Beyond the comparison with MSESC, MVGR-DMSC also demonstrates competitive performance against other state-of-the-art methods. On the Cora dataset, our model achieves the best results across all three metrics, outperforming methods like CVCL, DDMVC, and MSCIB by a clear margin. On Wikipedia, while DDMVC leads in ACC, MVGR-DMSC achieves the highest NMI and ARI, indicating better cluster purity and consistency. On the image side, our method delivers the best ACC and NMI on Caltech101_20, and ranks second on Handwritten, closely behind DDMVC.

Further analysis of the results shows different performance patterns across evaluation metrics. On the Wikipedia dataset, although DDMVC shows a higher ACC, MVGR-DMSC achieves better results in NMI and ARI. This difference stems from the characteristics of the Wikipedia data, which contains high-dimensional noise. While ACC measures the direct mapping to labels, NMI and ARI are more robust indicators of the underlying cluster structure. The higher NMI and ARI scores suggest that the dual-order graph regularization helps discover more consistent cluster manifolds, even when direct label matching is difficult. Similarly, for the Caltech101_20 dataset, MVGR-DMSC leads in ACC and NMI, while DDMVC performs better in ARI. This is likely because ACC and NMI emphasize global cluster assignments, whereas ARI is more sensitive to pair-wise relationships. Given the large number of clusters and high intra-class variance in Caltech101_20, our model shows a stronger capability in maintaining global category consistency.

Overall, the extensive experimental comparisons demonstrate that MVGR-DMSC not only significantly outperforms its baseline MSESC—with particularly dramatic improvements on challenging datasets like Wikipedia—but also achieves highly competitive results against a broad range of recent deep multi-view clustering approaches. This confirms that our presented innovations successfully enhance representation quality and clustering performance.

## Model analysis

### Ablation study

To assess the impact of each component in MVGR-DMSC, we evaluate the impact of the base self-expression loss, the dual-order graph regularization, and the deep clustering loss separately. The overall objective consists of three primary parts: the base self-expression term ${ℒ}_{base}=\frac{1}{2}\gamma {ℒ}_{rec}+{ℒ}_{enet}$, the graph regularization ${ℒ}_{graph}$, and the deep clustering term ${ℒ}_{cluster}$. We conduct ablation experiments on the ACM and Handwritten datasets by including or excluding certain loss terms, leading to four configurations, which are summarized in Table 5.

- **Base configuration (*M*_1_):** We first train the model using only the base reconstruction loss and elastic net regularization, serving as a baseline. The outcomes from this setup (*M*_1_) confirm the capability of the fundamental self-representation learning.
- **Adding graph regularization (*M*_2_):** Here, we incorporate the dual-order graph regularization term into the base loss. Relative to *M*_1_, including ${ℒ}_{graph}$ leads to better clustering metrics. This indicates that leveraging local manifold structures helps the model produce higher-quality representations.
- **Adding deep clustering loss (*M*_3_):** This configuration removes the graph regularization but includes the deep clustering loss. Compared to the baseline *M*_1_, the distribution alignment guided by clustering effectively encourages features to be more compact and cluster-friendly.
- **Full model (*M*_4_):** Finally, *M*_4_ combines all three loss components. The results from this full model demonstrate that our method successfully captures both complex geometric structures and intrinsic clustering distributions in multi-view data, yielding a substantial improvement in clustering performance.

**Table 5:** Ablation studies on loss components. Bold denotes the best results.

	Components			ACM			Handwritten		
	${ℒ}_{base}$	${ℒ}_{graph}$	${ℒ}_{cluster}$	ACC	NMI	ARI	ACC	NMI	ARI
*M*_1_	✓			0.4724	0.3630	0.2937	0.7160	0.8399	0.7394
*M*_2_	✓	✓		0.4767	0.3613	0.3084	0.7740	0.8610	0.7837
*M*_3_	✓		✓	0.4688	0.3597	0.3094	0.7495	0.8597	0.7672
*M*_4_	✓	✓	✓	**0.4952**	**0.3772**	**0.3244**	**0.7840**	**0.8758**	**0.8112**

Table 5 summarizes the outcomes of our ablation study. Relative to the baseline *M*_1_, including the dual-order graph regularization (*M*_2_) generally boosts clustering metrics, suggesting that preserving high-order manifold structures contributes to more robust representations. Similarly, adding the deep clustering loss (*M*_3_) aids in learning more cluster-friendly features. Finally, the full model *M*_4_ attains the highest scores on all metrics for both datasets. This confirms that the graph regularization and deep clustering guidance work synergistically. Through their joint optimization within a single framework, our method comprehensively leverages global, local, and clustering-structure information inherent in multi-view data.

### Parameter analysis

In the proposed MVGR-DMSC model, the overall objective function comprises two essential trade-off parameters, $\lambda_{1}$ and $\lambda_{2}$, which balance the loss components. Specifically, $\lambda_{1}$ governs the influence of the dual-order graph regularization in maintaining the local manifold structure, while $\lambda_{2}$ determines the weight of the adaptive view-weighted deep clustering loss to guide the representation learning. To thoroughly analyze how sensitive our model is to these hyperparameters, we perform a set of comprehensive parameter analysis experiments.

**Fig 2:**
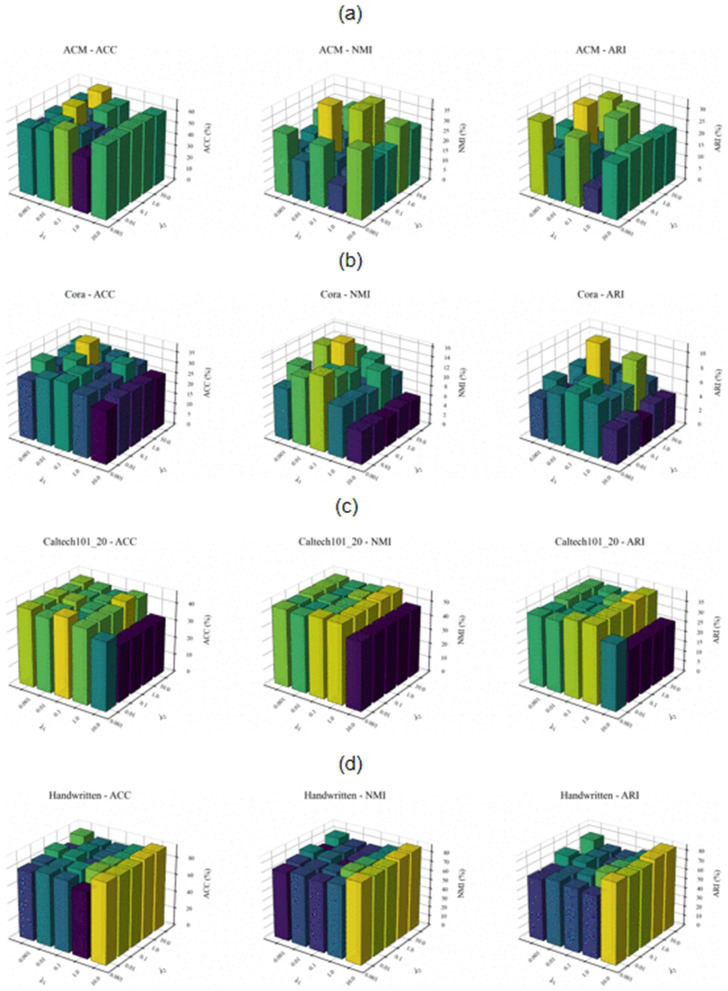
Parameter sensitivity analysis of $\lambda_{1}$ and $\lambda_{2}$ on four representative datasets.

We utilize a grid search strategy to identify the optimal parameter values. Specifically, we vary both $\lambda_{1}$ and $\lambda_{2}$ within the range of $\left\{10^{-3},10^{-2},10^{-1},10^{0},10^{1}\right\}$, while keeping other parameters fixed. Since the hyper-parameter setting strategies are consistent across all datasets, we select two representative text datasets (ACM and Cora) and two image datasets (Caltech101_20 and Handwritten) for visualization. The clustering performance variations in terms of ACC, NMI, and ARI are visualized in Fig 2.

As shown in the 3D bar plots, MVGR-DMSC exhibits remarkable stability across a broad spectrum of parameter combinations. For example, on the Handwritten dataset, the performance metrics exhibit a relatively flat plateau, demonstrating the model’s strong robustness against parameter variations. Although slight fluctuations can be observed on the ACM and Cora datasets when the parameters are set to extreme values, the overall clustering accuracy does not suffer from drastic degradation. This phenomenon explicitly demonstrates that MVGR-DMSC is insensitive to the specific choice of $\lambda_{1}$ and $\lambda_{2}$. Consequently, our model possesses strong robustness and stability, making it easy to tune and applicable to various real-world scenarios.

### Convergence study

To examine the convergence behavior of our optimization strategy, we record the values of the overall objective function (Eq. (18)) against the number of epochs across all five datasets. The training process is carried out strictly following the steps outlined in Algorithm 1.

**Fig 3:**
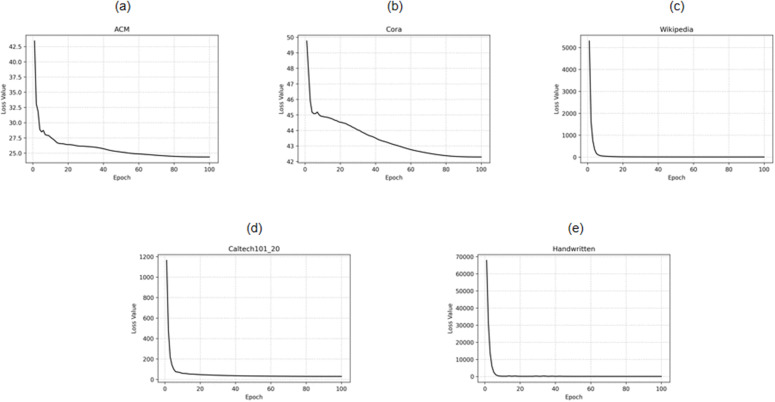
Convergence curves of MVGR-DMSC on five benchmark datasets over 100 epochs.

Fig 3 illustrates the convergence curves for ACM, Cora, Wikipedia, Caltech101_20, and Handwritten datasets over 100 epochs. As shown in the figures, the loss values across all datasets decrease rapidly during the initial training phase (the first 10–20 epochs). Subsequently, the curves gradually smooth out and stabilize at a fixed value after approximately 80–100 epochs.

Specifically, for datasets such as Handwritten and Wikipedia, the loss drops sharply, exhibiting an “L-shaped” curve, indicating the high efficiency of our gradient-descent optimization. For datasets with smaller loss magnitudes, such as ACM and Cora, although the visual scale differs, the downward trend remains continuous and eventually reaches a steady state. These results empirically demonstrate that the proposed Algorithm 1 exhibits excellent convergence properties and efficiently optimizes the network parameters Θ and the affinity matrix **C** to obtain a locally optimal solution.

### Visualization analysis

**Fig 4:**
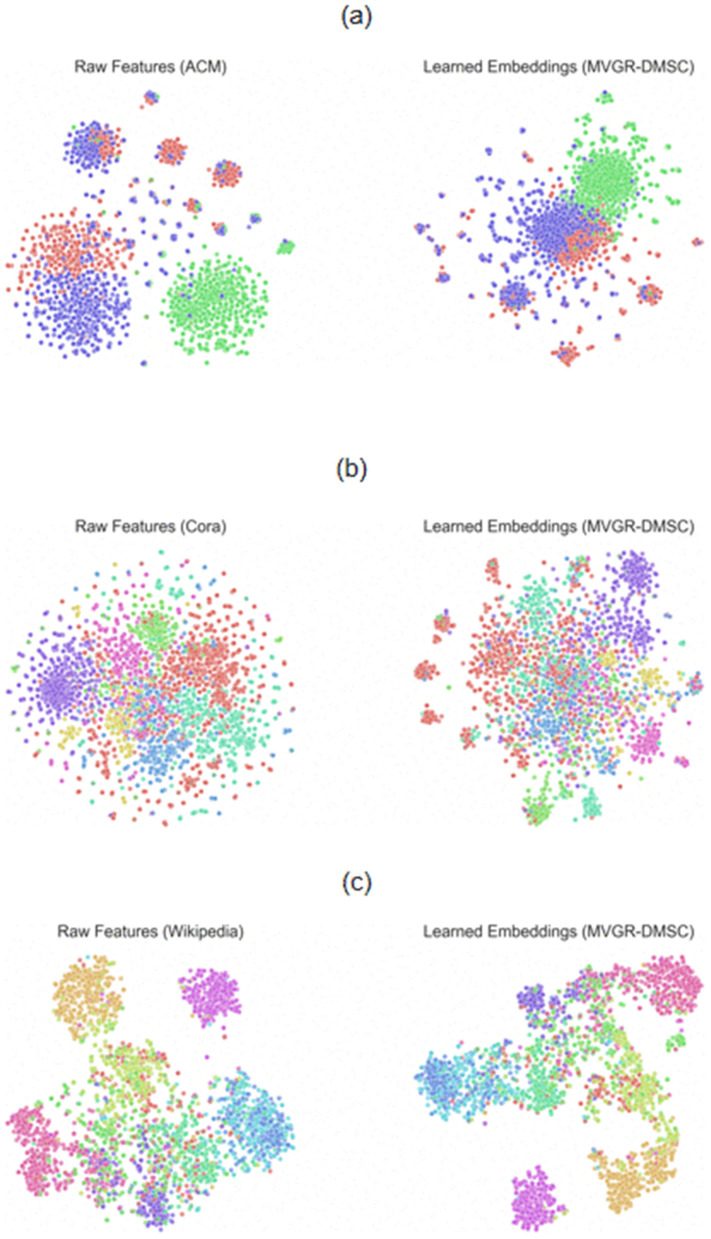
t-SNE visualization of raw features (left) and learned embeddings (right) on three benchmark datasets.

To gain a more intuitive insight into how MVGR-DMSC refines the feature space, we employ t-SNE to project the high-dimensional representations onto a 2D plane. Fig 4 presents the visual results on ACM, Cora, and Wikipedia datasets, comparing the distribution of concatenated raw features against the latent embeddings learned by our model.

As evidenced in the original feature spaces (the left panels of Fig 4(a)-4(c)), the data points appear as highly overlapped and entangled clouds. This lack of discriminative structure inherently limits the performance of traditional clustering methods. In contrast, the embeddings produced by MVGR-DMSC (the right panels) exhibit a significantly more organized topology. One can clearly observe that samples belonging to the same category are pulled into compact, high-density groups, while the margins between different clusters are substantially widened.

Specifically, the transition from a scattered distribution to a well-separated one in Fig 4(b) and 4(c) confirms that our dual-order graph regularization successfully captures the underlying manifold structure. Furthermore, the adaptive clustering guidance effectively filters out cross-view noise, allowing the model to transform nearly indistinguishable feature sets into distinct, identifiable clusters. These visual results provide strong empirical evidence that MVGR-DMSC successfully overcomes the limitations of traditional methods by learning highly discriminative representations. Ultimately, this demonstrates the superiority and efficacy of our presented model in handling complex multi-view clustering tasks.

## Conclusion

In this paper, we introduce a straightforward yet comprehensive framework termed Multi-View Graph Regularized Deep Metric Subspace Clustering (MVGR-DMSC). Unlike current deep multi-view clustering approaches that depend on a computationally expensive self-expression layer, our method directly produces the intrinsic similarity between soft assignments to guide representation learning and adaptively weights the contributions. We integrate dual-order graph regularization and adaptive view-weighted deep clustering into a unified framework. The dual-order graph regularization effectively preserves both local and high-order manifold structures, while the deep clustering module utilizes the KL divergence of soft assignments to guide the representation learning and adaptively weights the contribution of different views.

Extensive experiments on five benchmark datasets demonstrate that MVGR-DMSC significantly outperforms several state-of-the-art methods. The ablation studies and parameter analysis further verify the effectiveness of each component and the robustness of the proposed model. However, the current framework has some limitations. First, although the training process is efficient, the initial construction of dual-order graphs still incurs $𝒪(N^{2})$ complexity, which may limit its application to ultra-large-scale datasets. Second, the model assumes that all data views are complete, whereas missing views are common in real-world scenarios. Future work will focus on developing anchor-based graph construction strategies to further improve scalability and extending the framework to accommodate incomplete multi-view data. We also plan to explore automated parameter tuning mechanisms to reduce the reliance on manual grid search.

## Supporting information

The datasets and algorithms in this paper are all available at https://github.com/AVAVAdeep/MVGR-DMSC.
